# From Deutsche Zeitschrift to International Journal of Legal Medicine—100 years of legal medicine through the lens of journal articles: Part 1: *Deutsche Zeitschrift für die gesamte gerichtliche Medizin* from 1922 to 1944

**DOI:** 10.1007/s00414-022-02888-w

**Published:** 2022-09-30

**Authors:** Ingo Wirth, Tony Fracasso, Heidi Pfeiffer, Andreas Schmeling

**Affiliations:** 1 Berlin, Germany; 2 University Center of Legal Medicine (CURML), Geneva University Hospital, Geneva, Switzerland; 3 Institute of Legal Medicine, University Hospital Münster, Münster, Germany

**Keywords:** History of legal medicine, Official publications, Academic articles

## Abstract

From its launc h in 1922 to the end of the Second World War, the *Deutsche Zeitschrift für die gesamte gerichtliche Medizin* spanned 38 volumes. The 1762 papers contained in those volumes reflect contemporary interests and include many papers from peripheral fields and non-medico-legal disciplines. Publications concerned with issues outside core legal medicine fields in particular allow two distinct tendencies in the development of German institutes of legal medicine to be discerned. Firstly, there is a focus on the psychological and psychiatric aspects of the discipline. Secondly, there is tendency towards a scientific-criminalistic outlook. The fatal consequences of the Nazi seizure of power in 1933 did not spare the sciences. For legal medicine, a discipline with close links to the state, it is unsurprising that fundamental changes to the political system had a significant impact on subject matter. Leaving aside articles notable principally for their ideological content, our analysis of the 38 volumes shows that the papers examined contain new insights into many subjects, some of which are still valid today.

## Introduction

The International Journal of Legal Medicine can trace its roots right back to the middle of the nineteenth century. The journal which eventually developed into the International Journal of Legal Medicine was founded by Johann Ludwig Casper, the great reformer of Prussian legal medicine [1], who embodies the beginnings of the journal’s long tradition. He inaugurated and edited the *Vierteljahrsschrift für gerichtliche und öffentliche Medicin* (Quarterly Journal of Legal and Public Health Medicine), first published in early 1852. By the time of his death in 1864, a total of 25 volumes had been published. While a number of similar periodicals came and went [2], his *Vierteljahrsschrift* endured. New editor Wilhelm Horn praised the previous volumes as “an archive for science” in which “many of the most significant questions have been debated and brought nearer to a conclusion” [3]. The subsequent “*Neue Folge”* (New Series) commenced in 1864 with Volume 1 and was published under the existing title until Volume 15 in 1871, and thereafter as the *Vierteljahrsschrift für gerichtliche Medicin und öffentliches Sanitätswesen* (Quarterly Journal of Legal Medicine and Public Health) until Volume 53 in 1890. Following Horn’s death in 1871, from volume 14 the *Vierteljahrsschrift* was edited by Hermann Eulenburg. When Albrecht Wernich became editor in 1891, the “*Dritte Folge*” (Third Series) again restarted from Volume 1. From volume 12 in 1896, the appointment of Fritz Strassmann, associate professor and director of the Berlin institute, meant that, for the first time in decades, the journal was once more under the editorship of a representative of the legal medicine profession. He co-edited the journal with a senior government medical officer until it ceased publication with Volume 62 in 1921. The collaboration between two editors from different specialities helped to establish the *Vierteljahrsschrift* as a periodical for the old *Staatsarzneikunde* (combination of Legal Medicine and Public Health).

The *Dritte Folge* was directly followed from 1922 by the *Deutsche Zeitschrift für die gesamte gerichtliche Medizin* (which, for brevity, we hereafter refer to as the *Deutsche Zeitschrift*). The continuity between the two periodicals was made explicit by its subtitle, *Fortsetzung der Vierteljahrsschrift für gerichtliche Medizin und öffentliches Sanitätswesen* (Continuation of *Vierteljahrsschrift*). The title page also identified the new journal as *Organ der deutschen Gesellschaft für gerichtliche und soziale Medizin* (Official Journal of the German Association for Legal and Social Medicine), so that the scientific association [4], founded in 1904, now had its own journal. The expanded editorial board consisted of Fritz Strassmann, two of his postgraduate students, Paul Fraenckel based in Berlin and Georg Puppe based in Breslau, and forensic psychiatrist Ernst Schultze based in Göttingen. After Puppe’s death in 1925, Ernst Ziemke, a Professor at Kiel and also an adherent of the Strassmann school, joined the committee starting with Volume 7. Following the Nazi seizure of power, two further editors—Friedrich Pietrusky, a professor from Bonn and Eduard Schütt, a legal medical officer from Wuppertal-Barmen—joined the editorial board commencing with Volume 21. Both had distinguished themselves through their exceptionally zealous support for the new ruling party [5]. In 1935, the worsening political situation in Germany resulted in a reorganisation of the editorial board. The implementation of the Reich Citizenship Law, the Nazi law depriving Jews of their German citizenship, meant that Fraenckel from Volume 25 and shortly afterwards Strassmann from Volume 26 were removed from their positions—and not just at the journal [6]. At the same time, Ziemke’s death in 1935 reduced the number of editors further, with Hermann Merkel from Munich appointed only in 1938 from Volume 29. Schultze died that same year, but the editorial board did not appoint another forensic psychiatrist to replace him. The *Deutsche Zeitschrift* continued to be edited by Merkel, Pietrusky und Schütt until Volume 35 in 1942. Pietrusky was then replaced by Kurt Walcher from Würzburg for Volumes 36 to 38 until 1944. The last issue of the *Deutsche Zeitschrift* to be published before the war’s end was Issue 5 of Volume 38 in spring 1944.

As in the previous quarterly journal, after 1922 the contents continued to span original papers, abstracts and reports. As the official journal of the scientific association, the *Deutsche Zeitschrift* was used to publish conference proceedings. Our analysis focuses on original papers, because our aim is to present the academic development of legal medicine and related fields through the lens of the *Deutsche Zeitschrift*. We have therefore considered both original papers and presentations from the proceedings of the scientific association prepared for publication. In addition, for our exploration of the history and development of the subject, we have also considered opening speeches from conferences.

## Analysis of academic articles

### Key areas of academic enquiry in the period from 1922 to 1944

In keeping with the broad-based concept underpinning the *Deutsche Zeitschrift*, which extends well beyond the narrow confines of legal medicine, in addition to core legal medicine topics, our analysis of the content also covers topics from related forensic science fields. Other topics covered include historical and epistemological matters, plus laudations, obituaries, etc. The classification on which the analysis is based and an overview of the number of publications on individual topics over the period under review is shown in Table 1.

**Table 1:** Overview of the number of publications on individual topics in the period 1922 to 1944

Topic	Number of publications
History and evolution of legal medicine	
History of the discipline Evolution of the discipline	4 25
Personalia (People)	33
Legal issues Expert witness work	90 49
General legal medicine	
Post-mortem examination Thanatology Vital reactions	71 74 36
Forensic traumatology and pathology	
Forensic traumatology Forensic obstetrics Abortion Infanticide Medical malpractice Illness and sudden unexpected natural death	346 69 30 18 26 78
Forensic toxicology	
Clinical signs of poisoning Pathological anatomical findings Toxicological chemical analytical methods Toxicology of alcohol	125 36 68 64
Identification of unknown decedents	21
Forensic genetics	150
Scientific-technical criminalistics	113
Clinical legal medicine	7
Forensic psychiatry and psychology	75
Sexual medicine	57
Traffic medicine	19
Social medicine	11
Criminology	67
Total	**1762**

### History and evolution of legal medicine

Legal medicine is one of the oldest medical specialities. Its position on the boundary of medicine and law means that it has historical links to a wide range of other disciplines (24/1).^1^ The literature on the *history of legal medicine* does not reflect this variety. This deficiency, as he saw it, prompted Ferdinand von Neureiter to call for collaboration on a “History of German Legal Medicine as a Subject for Research and Teaching” (28/60).

The interdisciplinary nature of legal medicine means that, over the years, the *evolution of the subject* has often been closely scrutinised. In 1924, Willy Vorkastner spoke at length on “The Status and Role of Legal Medicine” (5/89). In two key articles from 1928, both entitled “Old and New Directions in Legal Medicine” (11/1, 11/14), Richard Kockel and Fritz Reuter explored the different directions in which the discipline was evolving.

The proceedings of the scientific association [4], which were printed in Volume 1 and later at irregular intervals in the *Deutsche Zeitschrift*, offer a useful overview of topical issues in legal medicine. Between 1933 and 1940, the journal published edited versions of talks at the 21^st^ and 24^th^ to 29^th^ congresses of the scientific association. On many occasions, these expert gatherings were accompanied by political declarations reflecting the zeitgeist. As chairman of the German Society for Legal and Social Medicine, Berthold Mueller, at the time a Professor in Göttingen, concluded his opening speech at the 26^th^ congress in spring 1937 (29/133) with the words, “As with all collective endeavours, today we would like to turn our thoughts to the man who, despite differences of opinion large and small, beyond parties and hatred, has brought the German people together to form a unified whole. We are conscious that, in our academic work too, we are answerable to him and to the German people. Heil Hitler!”.

The opening speech at the 28^th^ congress in spring 1939 (32/191) was given by Arthur Gütt, Undersecretary at the Reich Ministry of the Interior. He gave delegates a detailed elucidation of the Health Service Standardisation Law of July 3, 1934. The Law replaced district medical officers with Health Authorities which, under Sect. 3(1)(III) of the Law, were made responsible for all legal medicine activities. As one advantage of this reform, Gütt emphasised the close relationship between the role performed by legal medicine and “*Erb- und Rassenpflege*” (“genetic and racial hygiene”).

The welcome message delivered by Gerhard Buhtz, Chairman of the Society and a Professor at Breslau, at the 29^th^ congress in spring 1940 (34/1), was also driven by the prevailing zeitgeist. In conjunction with a declaration of loyalty to the Nazi leadership and the war goals, he discussed endeavours to link together legal medicine and forensic science. In keeping with this trend, papers on requirements for university teaching focus particularly on teaching for detectives. Endeavours to achieve effective collaboration between legal medicine specialists, forensic scientists and lawyers are a key topic in Volume 18. Articles range from the role of legal medicine specialists at crime scenes, to issues relating to training, to the practice of law enforcement.

### Personalia (People)

From the beginning, the *Deutsche Zeitschrift* served as the official journal of the scientific association. Leading personalities of the discipline were therefore celebrated on notable birthdays or in obituaries. In addition, a number of issues took the form of commemorative issues dedicated to Albin Haberda (1/581), Richard Kockel (5/3), Carl Ipsen (7/137), Ernst Ziemke (10/147), Fritz Strassmann (12/1), Emil Ungar (14/3) and Hermann Merkel (21/57).

### Legal issues, expert witness work

Within the category *legal issues*, the most popular subject by far is criminal law and criminal procedure. The largest group is made up of 13 papers from the 1920s, in which medics and criminal lawyers discuss the oft-revised draft of the *Allgemeines Deutsches Strafgesetzbuch* (German General Criminal Code). With respect to the General Part of the Criminal Code in force at the time, most notable are articles on guilt and criminal responsibility. The majority of discussions relating to the Special Part of the Criminal Code deal with clauses on violent and sexual offences. Of the papers concerned with criminal procedure, 3 of 7 papers deal with police interrogation. The period after 1933 sees the publication of 10 works explicitly referring to “Nazi criminal legislation”. There are two reviews exploring the realignment of criminal law principles. In one, a senior public prosecutor from Stendal states, “We absolutely may view the law as representing binding principles and at the same time an order from the government as to how things should be arranged. The legal system thus becomes a system of formal orders from the Führer. Criminal legislation in particular acquires meaning and purpose in the great work of the new concept of the ethnic body politic, which began with detoxification, purification and healing and progresses to defence and education” (24/8). The second review was penned by Berthold Mueller, who analysed some of the individual provisions of the future criminal law and criminal procedure. Medico-legal facets of his review include such problems as the principle of strict liability, whereby the harmful consequences of a criminal act are of less significance than the criminal will of the offender, *Rasseverrat* (“racial betrayal”), which asks forensic medical specialists to decide whether the identification of certain non-German characteristics in a child can be used to conclude with sufficient certainty that sexual intercourse with someone from “another race” has taken place, and, his final example, euthanasia, “The proposals set out in the Prussian memorandum [“Nazi criminal law”] would also make possible the destruction of *lebensunwertes Leben* (“life unworthy of life”). I do not believe any objections to this can be raised from either a racial or medical point of view” (24/114). Other articles on Nazi criminal law deal with other far-reaching new legislation, such as the *Gesetz zur Verhütung erbkranken Nachwuchses* (Law for the Prevention of Offspring with Hereditary Diseases) or the *Verordnung gegen Volksschädlinge* (roughly translatable as Regulation against Parasites on the Body of the People).

Articles on other areas of law are primarily concerned with family law and medical law. A review entitled “Legal Issues in Road Traffic Accidents” (28/30) deals with current legislation and case law. There are also isolated essays on the Code of Civil Procedure, the Prison Act and social legislation. Papers on civil law include an essay by Heinrich Többen on “Farmer Eligibility under the Reich Farm Inheritance Act” (29/317), a characteristic piece of Nazi legislation.

Papers on *expert witness work* can be divided into two groups. There is a general group comprising 10 articles on methodological problems in appraisals and on fundamental questions such as causality or expert witness professional confidentiality. There is also a more specific group of 39 articles concerned with the significance of medical opinions in specific questions. Most of the published expert witness reports deal with an assessment of the causal relationship between physical injuries and preceding trauma. Thirteen of these relate to insurance medicine, and there are a further 4 case reports on simulation. Three of the case studies on simulated symptoms of illness include descriptions of experiences from the First World War, which in some cases attained renewed significance in the context of insurance assessments.

### General legal medicine

The majority of papers on *post-mortem inspection and forensic autopsy* are concerned with the performance of these two basic post-mortem diagnostic techniques. Seven articles deal with domestic and foreign regulations on autopsy and the purpose and objectives of court-ordered forensic autopsies. A further 7 articles concern specific autopsy techniques. Additional investigations performed after autopsies span a wide spectrum of techniques and are accordingly found in a number of volumes. Most papers are concerned with either toxicology or histological examination. The 14 publications in this area include both microscopy techniques and forensically significant microscopy findings from histological examination of organ material. There are also 3 papers on bacteriological and 3 on biochemical analysis, 2 on X-ray examinations and 3 on a range of other techniques. Twelve papers discuss expert evaluation of autopsy results. Problems of competing causes of death and the capacity to act of the victim following various traumas are considered in a number of papers. Eight papers deal with exhumation, with repeated emphasis on its diagnostic value. Finally, in 1922 and 1936, several authors called for the introduction of coroner’s post-mortem examinations.

Twenty-seven papers, almost half of all papers on thanatology, are concerned with *early post-mortem changes*. The phenomenon of supravital reactions includes studies on early post-mortem pupil changes (6/22, 6/32, 20/144), on tissue changes as a result of autolysis (3/359, 16/61), and individual studies on the pattern (11/317) and colour (13/261) of livor mortis. There are 17 articles on problems relating to rigor mortis, forming the largest group of articles relating to thanatology. The main topic considered is the physiology of rigor mortis and the lactic acid theory (2/1). There are also earnest discussions of the special case of catalepsy in both a detailed literature review (2/647) and a collection of case studies (3/349). In addition, there are 3 reports on cases of cataleptic spasm (3/357, 3/562, 13/13). There are a total of 28 papers on *late post-mortem changes*. Dictated by practical concerns, the dominant theme is decomposition of the body, with 7 papers primarily concerned with differentiating between putrefactive gases and air embolism. A series of publications (4/562, 6/650, 9/459) on this diagnostic difficulty were published by Berlin forensic pathologist Felix Dyrenfurth, whose earlier findings were nonetheless overlooked in a later study (6/379). There is also a paper on gas analysis in putrefying lungs (16/459). Three papers explore the aetiology of coffin birth, which at the time had not been definitively explained. There are 9 papers on preservative post-mortem changes and 5 papers on animal bites on bodies. In the field of *thanatochemistry*, there are 6 papers on the production and detection of putrefaction products such as sulphhaemoglobin and biogenic amines.

The utility of individual post-mortem changes and the diagnostic value of stomach contents and stomach mucosa are discussed as methodologies for *time of death estimation*. A review summarising the current state of knowledge was penned by Hermann Merkel in 1930 (15/285). Still in the 1930s appeared the first papers on correlations between post-mortem rectal temperature and time of death (28/172, 29/158, 31/256).

With respect to the phenomenon of *vital reactions*, by far the largest number of papers are on general reactions. In particular there are 19 papers on embolism, with the largest number of papers on air embolism, followed by fat embolism, and finally tissue embolism. There is one review devoted to the medico-legal significance of these types of embolism when presenting as a paradoxical embolism (23/338). The general vital reactions described include individual studies on haemorrhage, shock, aspiration and swallowing. There is relatively little on local vital reactions. In addition to 2 papers on thrombosis (15/330, 21/147), there are a variety of post-mortem biochemical analyses intended to identify vital changes in post-mortem blood, muscle tissue, bronchi, nerve cells and cerebrospinal fluid.

### Forensic traumatology and pathology

Injuries from *blunt force trauma* are as varied as the mechanisms by which they are produced. It is not uncommon that external findings are able to shed light on the event that caused them. This is true for the shape of epithelial abrasions (37/33), the morphology of the wound margin (22/299) and “ischaemic impact patterns” (29/408). Scalp wounds also have practical diagnostic significance, and specific features of these wounds are presented in 3 papers. If we look at the distribution of violence by body region, records of head injuries are by far the most common. Of the 11 papers which focus specifically on the shape of skull fractures, one is concerned with the sequence of skull fractures (8/430) and one with the fracture mechanism in babies (32/461). The 30 papers on the wide variety of brain injuries range from concussion and intracranial haemorrhages to delayed consequences of brain injuries. The mechanism of coup-contrecoup injuries is the most frequent subject, discussed in 7 papers, with some debate over Lenggenhager’s centrifugal theory as a potential mechanism (31/61, 31/278, 31/280). There are also several papers on the consequences of blunt force to the trunk and extremities. The most common subject, with 12 papers, is blunt abdominal trauma and resulting injuries to the parenchymal organs and gastrointestinal tract. There are detailed discussions of the difficulties of reconstructing events in the context of a fall in the mountains (28/90) and a fall from height (30/334). Boxing-related deaths form a special category, with 6 case studies published. The causes of death were intracranial haemorrhage (12/392, 16/341, 25/41), one case of “death from shock”, in the sense of a sudden cardiac event (1/481, 19/415), and suffocation due to acute laryngeal oedema (1/695). Finally, a further example of death from blunt force is the legendary case report “A new method of insurance fraud: the Tetzner case” (21/112) by Richard Kockel. Of publications on the consequences of sharp force, by far the largest number are concerned with stab wounds. Almost all of the 21 papers on the subject deal with knife wounds and the possibility of using the morphology of the wound to draw conclusions on the implement used or the events of the crime. Far less common are cuts. Five of 6 case reports are concerned with the question of whether a death has occurred as a result of murder or suicide in cases where the throat has been slit. A further small group of cases consists of 5 papers on wounds inflicted with slashing weapons, in which once again the focus is on the question of whether the injuries were inflicted by the victim or a third party. In addition to 2 collections of case studies (2/412, 22/407), there are case reports on 3 suicides involving a blow from an axe (20/64, 27/308, 28/422). A special form of penetrating trauma is bite wounds. Two of 3 papers on this subject are concerned with the value of bite wounds for identification purposes (16/89, 29/453). There are 4 case studies on criminal corpse dismemberment with cutting tools, 2 of which deal with medico-legal aspects of serial murders by Karl Großmann (3/147) and Karl Denke (8/703). Other reports on high-profile murders committed using penetrating force include the widely reported sexually motivated murder of high school graduate Helmut Daube (14/158) and serial murders by sadist Peter Kürten (17/247).

There are 13 works on *asphyxiation*, including a number of unusual case studies and several experimental studies discussing morphological features diagnostic of asphyxiation. Gerhard Schrader penned a review (28/134) in which he attempts to produce a systematic overview of then (mid-1930s) known diagnostic criteria for violent asphyxiation. Six of the 7 reports on homicides through *manual strangulation* concern crimes where the perpetrator has attempted to disguise the crime. One further paper deals with the significance of the carotid sinus reflex (*Hering* reflex) in strangulation. Following a comparative analysis of human corpses and experimental animal corpses, the author expresses the opinion “that a powerful stranglehold around the neck can lead to unconsciousness as a result of the carotid sinus reflex, without leaving localised signs of strangulation on the victim” (20/361). There are only 5 articles on *ligature strangulation*, with 3 case reports highlighting the possibility of self-strangulation. A report on a homicide triggered an expert debate on the carotid sinus reflex during ligature strangulation (15/419, 15/572). There are 25 case reports on *hanging*. The most significant from a pathophysiological perspective are 3 extensive studies (1/686, 4/165, 11/145) on the mechanism of death by hanging. Further insights into the dying process are provided by “Observations on Death in Executions by Hanging” (22/192) from 1917/18.

The vast majority of papers on *death in water* are concerned with drowning. These include 8 papers on the pathophysiology of drowning and 3 on morphological findings. Erich Fritz describes a new sign of death by drowning in the form of tears in the mucous membrane of the stomach in people who have died by drowning (18/285), and Gyula Balázs’ collection of case studies represents the first report on “ischaemic impact patterns” after falling into water (21/515). Nine papers describe microscopic and biochemical techniques for diagnosing death by drowning. There are 11 papers concerned with post-mortem changes in bodies recovered from water and the phenomenon by which a body will float back up to the surface some time after death. There are individual treatises on the causes of atypical drowning and death when diving. An instructive overview of death by drowning (18/557) was produced by Gerhard Buhtz.

Investigation of *gunshot wounds* necessitates close cooperation with forensic investigators, with the result that the section on ballistics includes a number of such publications from the forensic science field. From a medico-legal perspective, the focus is on the effects of and expert assessment of gunshots in humans. A key diagnostic objective is recognising and distinguishing the entry and exit wounds. There are 17 papers on this topic. With respect to determining the range of fire, there are 13 articles on signs indicative of gunshot from close range, including Anton Werkgartner’s first paper in the *Deutsche Zeitschrift* on muzzle imprint in contact wounds (11/154). There is just one paper (23/375) on determining the direction of fire from the body. In terms of body region, the vast majority, 18 papers, deal with specific features of gunshot wounds to the skull, including one case of a Krönlein shot (1/141). There are just 5 articles on gunshots passing through the chest, causing fatal injuries to the heart and lungs. There are 10 case reports on homicides involving firearms, including an interpretation of findings relating to the shots that killed Communist Party of Germany co-founder Karl Liebknecht (5/247). From 1940, there are 3 extensive reports on large-scale investigations into multiple gunshot wounds. Two legal medicine specialists working separately detail their findings in relation to the victims of Bloody Sunday in Bromberg (34/7) and “from the hostage processions in the Warthegau” (34/54). A forensic science approach is taken in reporting the results of an “Investigation into Polish Atrocities against Ethnic German” (34/90). Seven papers describe rarely used weapon systems (animal stunning equipment, shotguns, terzeroles, Karabiner 98 k) and the characteristic wounds they produce. Finally, 3 reports on explosion injuries can also be considered to belong to this group of topics. These include just a single study on bomb and shrapnel injuries from hostilities during the Second World War (35/173).

There are 16 papers on *death from electricity*, encompassing accidents, suicides and homicides. There are also articles on non-fatal electrical injuries and on animal experiments to investigate electricity-specific effects. There are 5 papers on the appearance and identification of electrical burn marks from the electrothermal effect on the skin. The state of knowledge at the time is summarised in a review by leading expert Stefan Jellinek “On Electropathological Semiotics and Causality” (12/104) and by Friedrich Pietrusky on morphological findings after exposure to technical electricity (29/135). There is also a collection of case studies (32/407) concerned specifically with the consequences of lightning strike.

Thermal damage caused by *heat*, with a focus on morphological findings in burns victims, is the subject of 5 publications. Hermann Merkel published a survey of diagnostic options for burnt corpses (18/232). Of the other publications, there are 3 papers on microscopy findings in human corpses (19/293, 23/19, 23/281) and one study using animal experiments (20/445). There is also one paper on delayed death from burns (35/75) and one on the pathogenesis of scalding (3/401). In addition, there is a case report on a self-scalding by a psychiatric patient in a boiling pan (36/49). A collection of case studies discusses 3 homicides by burning (21/120). The disposal of corpses by burning is the subject of a further 2 papers, and there is also an article entitled “Self-immolation of the Human Body” (18/437) which takes a historical criticism approach. Two papers deal with injuries caused by *cold*. One reports on the clinical progression of fatal hypothermia (18/270), while the other reports on results from animal studies on general hypothermia (30/199).

Physical changes in the corpse after *starvation* are described in detail in the context of a death in connection with a fasting regime (6/520).

Reports on single or multiple deaths involving *various types of external violence* are not easily assigned to any of the above groups. These 9 papers are therefore recorded separately. In addition there are two collections of case studies on fatal sport-related accidents occurring in the course of a wide range of different sport activities. Table 2 shows a summary of the number of publications on all types of violent death.

**Table 2:** Articles on violent death from 1922 to 1944

Violent death	Number of publications
Blunt force	86
Sharp force	41
Asphyxiation	51
Death in water	35
Gunshots	77
Electricity	30
Heat	12
Cold	2
Starvation	1
Multiple violence	11
Total	**346**

*Forensic obstetrics* as a speciality is concerned with forensic medical problems relating to pregnancy and childbirth and to the physical condition of neonates. The first group of 14 papers deals with health risks posed by mechanical contraceptive devices, pregnancy testing, complications of pregnancy and indications for terminating pregnancy. Eleven articles on complications of childbirth affecting mother or child are primarily focused on reliably distinguishing spontaneous injury from the results of criminal acts. Six papers on equivocal findings in neonates concern the same objective. Section 90 of the German Code of Criminal Procedure (StPO), which sets out the regulations on autopsies on neonates, raises several questions for the pathologist performing the autopsy. These are discussed in a total of 38 publications. One recurring theme is determining whether an infant was alive at birth, whereby in addition to a number of papers on the lung float test (2/31, 2/267, 5/47, 6/5, 14/7), a cord vessel test (36/21) is cited as a novel method for answering this question. The then Sect. 218 of the German Criminal Code (StGB), which dealt with termination of pregnancy, meant that from the 1920s to the 1940s *abortion* was one of the primary issues in forensic obstetrics. Consequently, the 30 publications on the methods and consequences of illegal procedures on pregnant women represent a further major topic for the volumes of the *Deutsche Zeitschrift* examined by this review. While doctors were motivated to oppose illegal abortion because of the health risks for the pregnant women, the Nazi era saw the focus of opposition shift to population policy objectives. Thus we have a 1940 publication entitled, “The Fight against Abortion as a Political Task” (32/226). There were 18 publications on *infanticide* as defined in Sect. 217 of the German Criminal Code (StGB). These illustrate the difficulties of forensic diagnostics, which consisted primarily of using morphological features to distinguish maternal attempts to facilitate delivery from actions performed with the intention of killing the child.

Publications on *medical malpractice* are concerned with medical interventions in both surgical and non-surgical specialities. The most common topic is reports on adverse drug events, of which there are 10 cases. There are 4 reports on anaesthesia-related adverse events during surgery and an equal number alleging post-operative medical errors. There are also 3 case reports on radiation injuries, 2 on complications during diagnostic procedures and 2 on vaccine-related injuries. There is also a review tackling the subject of the “History and Concept of Malpractice” (20/161), primarily from a legal perspective.

With respect to papers on disease and *sudden unexpected natural death*, there is a dramatic imbalance in the number of cases concerning the two age groups generally analysed. While there are 65 publications on adults, there are only 13 papers on sudden unexpected natural death in children. The most frequently cited groups of fatal diseases in adults are diseases of the central nervous system, on which there are 15 publications, and cardiovascular disease with 13 articles. There were far fewer reports on other groups of fatal diseases. There are 3 reports on deceased children, each of which describes malformations or infections as the cause of death. Other publications on childhood consist of individual case reports on children with rare diagnoses and reports on experience gleaned from large numbers of autopsies.

### Forensic toxicology

The largest group of publications in this subject area consists of descriptions of the *clinical features of various types of poisoning*, on which there are 125 papers. In addition to 9 collections of case studies on various poisons and 3 cases of combined poisoning, there are 68 papers on inorganic poisons and 45 on organic poisons. Of the inorganic poisons, by far the most widely discussed are carbon monoxide, on which there are 13 papers, arsenic, on which there are 9, and thallium, on which there are 7. Of the organic poisons, there are notable clusters of 17 papers on plant toxins and 13 reports on pharmaceutical poisoning. With respect to illegal drugs, widespread today, there is just one article on the suicide by cocaine of a male addict (4/40) and one on fatal poisoning with heroin after being mistaken for cocaine by a cocaine-dependent individual (12/112).

The identification of *pathological changes in tissue and organ anatomy* plays a role in the diagnosis of poisoning, though it does have some natural limitations. Of 28 papers on this subject, primarily on organ toxicity, 13 are concerned with the central nervous system, which is particularly vulnerable to poisoning. There are also 2 articles on characteristic morphological changes in specific types of poisoning. These are Mees’ lines and coma blisters, both first described in the *Deutsche Zeitschrift* (34/307). The second, smaller group on pathological anatomical changes consists of 8 reports on histological changes in various organs caused by one or more poisons.

The gold standard for diagnosing poisoning is *toxicological analysis*. In the period under review, 68 papers were published on this subject, of which 59 are concerned with the detection of a single poison or group of substances. As might be expected given the prevalence of carbon monoxide poisoning in that era, by far the most common, with 16 examples, are papers on various techniques for detecting CO or CO-Hb. Of groups of substances, alkaloid detection is described in 4 papers. In contrast to analysis of specific substances, there are 9 papers on the technical details of a variety of analytical methods, including spectrophotometry (1/411) and microscopy-based melting point determination (34/353).

One area of toxicology which has evolved into its own extensive speciality is the *toxicology of alcohol*. Of 33 publications on alcohol metabolism, 26 studies are concerned with factors which might affect the absorption, distribution and elimination of alcohol, such as age, trauma and chemicals. Just 5 publications focus on the correlation between blood alcohol concentration and the effects of alcohol. These include a review entitled “On the Terminology and Forensic Assessment of Alcoholic Intoxication” (14/296), which outlines the state of knowledge in 1930. The only paper on fatal alcohol poisoning (33/44) is primarily concerned with developmental peculiarities of alcohol clearance in children. There are 2 papers specifically on methanol poisoning. Thirty-one publications deal with methods for determining alcohol concentration in body fluids and tissues. In addition to two reviews (10/377, 11/134), there are papers on individual methods, including the Nicloux method (18/638) and the Widmark method (19/513). Between these papers and the end of the war, there are 18 further studies on Widmark’s micromethod. All of these papers deal with subtle technical points for overcoming shortcomings in the method and eliminating sources of error.

### Identification of unknown decedents

There are a total of 21 publications on then common medico-legal *identification methods*. Six publications in the field of osteology discuss ways of differentiating between species, sex diagnosis and age estimation from skeletal remains. One of these papers, which takes a detailed look at the forensic medical significance of the humerus, includes basic data on several identification procedures (22/332). There are also 4 papers specifically on skull identification, 2 of which include detailed descriptions of techniques for the superprojection of an unknown skull onto a photograph of a missing person (20/33, 27/335). Five papers describe odontological features which can be used for identification, and 2 linked articles discuss identifying features visible using radiological techniques. A case study on the particular utility of the frontal sinuses for identification (7/625) is the first paper on X-ray comparison in the German-language literature (10/81). There are 2 papers dealing with methods for specific investigatory queries and combinations of methods required for problem cases.

### Forensic genetics

Table 3 shows a summary of the number of publications on forensic genetics. The papers on the topic of blood groups cover test methods, prevalence, distribution and inheritance. The first paper on *blood groups* to appear in the *Deutsche Zeitschrift* was published in 1924 and was entitled “On the Forensic Significance of Isoagglutination of Red Blood Cells in Humans” (3/42). The papers on test methods discuss the production, use and storage of test sera and other reactants. The first publication on the prevalence and distribution of A subgroups in the *Deutsche Zeitschrift* was by Karl Landsteiner, the discoverer of the main blood groups. Another pioneer of blood group research, Berlin serologist Fritz Schiff [7], published the first paper on “The Medico-legal Significance of Landsteiner and Levine’s M and N Serological Properties” (18/41). Seventeen further articles on the M, N and P blood group systems followed. The publications on the inheritance of blood groups deal with Bernstein’s theory and of the inheritance model devised by von Dungern and Hirszfeld. There is also one experimental study on the secretion of blood group substances (28/234), the results of which confirmed that secretor status is inherited in a dominant manner.

**Table 3:** Articles on forensic genetics from 1922 to 1944

Topic	Number of publications
Blood groups	
Test methods	9
Prevalence and distribution	32
Inheritance	3
Trace evidence analysis	
Blood detection	4
Species specificity determination	13
Individuation	38
Bodily fluid traces	7
Hair traces	9
Faeces traces	2
Family relationship evaluation	
Fertility testing	1
Period of gestation	1
Heriditary biology	11
Blood groups	20
Total	**150**

The practical use of identificatory blood groups is the subject of medico-legal *trace evidence analysis*. The contemporary arsenal of methods necessitated a hierarchical sequence of examinations: test for the presence of blood − determine species specificity – determine blood group. Four articles describe peroxidase and crystal tests for *blood detection*. The specificity of these tests varies and depending on the test method should be considered a presumptive test only. The Uhlenhuth test has been used to *determine species specificity* since 1901, predominantly to distinguish whether trace material is human or animal [8]. The precipitation principle is the subject of 11 publications, and there are a further 2 papers on alternative serological methods for differentiating between species.

The discovery of human blood groups was soon followed by the development of the first methodical approach to the *individuation of bloodstains*. Landsteiner and Richter’s agglutination test represents the beginning of the use of inherited blood groups for forensic purposes [9]. The small quantities and ageing of trace material require modifications to test techniques. To achieve optimum results under these conditions, Leone Lattes introduced the coverslip method (9/402) and Franz Josef Holzer further refined Schiff’s agglutinin binding test [10] (16/445). As the publications on this topic show, of the then known blood group systems, forensic trace evidence analysis determined both AB0 system and MN group groups. Other publications deal with specific trace evidence-related issues. These include detecting menstrual blood by using its inhibitory effect on lupin seedlings and on yeast fermentation, sex diagnosis of bloodstains using colour reactions, using solubility and enzyme activity to evaluate the age of traces and using colorimetry to estimate the amount of blood.

Detection methods based on characteristic constituents of bodily fluids were developed for various *bodily fluid traces*. Epithelial glycogen content can be used to identify vaginal secretions (4/1). There are 4 papers on sperm detection, including a new microscopy-based detection method using Baecchi’s stain (27/143). There is also a review describing a number of older tests for detecting dried saliva (11/211). At the time, it was possible to determine AB0 groups both from semen and from other bodily fluids (23/186).

The majority of papers on *hair traces* is concerned with using morphological features for species differentiation. Other topics include determining developmental stage and sex, and demonstrating damage to the hair.

The papers on *traces of faeces* discuss serological identification based on coliform bacteria and a presumptive assignment of blood group.

In the period under review, *family relationship testing* relied on fertility testing, assessment of the period of gestation, a comparison of morphological characteristics and blood typing. There is one paper on fertility testing (26/64) and one on the legal and medical problems involved in assessing the period of gestation (14/11). The papers on hereditary biology deal with various physical characteristics for assessing similarity and with dactyloscopic criteria. By far the largest number of articles are on the results of blood group testing. As well as helping to clarify cases of disputed paternity, blood group analysis also served as evidence in a case of babies switched at birth (25/79). There is also a report by a veterinarian on “Blood Groups and Paternity Determination in Horses” (25/231).

### Scientific-technical criminalistics

There are numerous areas of overlap between legal medicine and scientific-technical criminalistics. In the period under review, these went well beyond the kindred forensic biology role performed by the two disciplines. Table 4 shows a summary of the number of publications on scientific-technical criminalistics.

**Table 4:** Articles on scientific-technical criminalistics from 1922 to 1944

Topic	Number of publications
General topics	2
Theory of forensics	1
Forensic biology	16
Forensic chemistry	15
Ballistics	33
Writing examination	15
Document examination	3
Dactyloscopy	10
Shape traces	8
Photography	4
Odorology	1
Investigative techniques	5
Total	**113**

In his article “Institutes of Legal Medicine and Criminalistic-Technical Activities Performed within Them” (14/411), Martin Nippe explicitly advocates for legal medical institutions to take over criminalistic tasks. His argument that the courts and police lacked the necessary expertise seems entirely reasonable in light of advances in forensic technology in Germany at the time. The urgency of this need is demonstrated by a collection of case studies from Göttingen College of Legal Medicine (14/26). It features cases which indisputably fall within the purview of forensic technology.

One field in which there were particular deficits was *theory of forensics*, on which only a single paper (34/404) was published. This paper, by distinguished Berlin criminalist Hans Schneickert, deals with the key insight that the prevalence of an identifying feature determines its value for identification purposes.

The picture is very different for the science-focused sub-disciplines of forensic technology. Almost exclusively as a result of research performed at institutes of legal medicine, a considerable body of knowledge had already been amassed in the field of *forensic biology*. In Germany, this sub-specialism was not an established academic discipline in the period under review, so that in the majority of cases the investigation of biological trace evidence was contracted to institutes of legal medicine. Accordingly, the theory behind many of the methods used in forensic genetics is equally applicable in the field of forensic biology. Twelve publications relate to the key criminalistic task of finding and securing bloodstains at a crime scene and on objects. Two studies on the morphology of bloodstains deal with fundamental insights into blood splatter patterns (16/272) and drip patterns (22/387). A further 2 papers are concerned with non-human material.

Of the publications on *forensic chemistry*, there are 8 papers on chemical analysis for the purpose of determining the cause of fire. In relation to 2 attempted fatal poisonings, there is a report on demonstrating the presence of poison in food. In addition, there are papers on the chemical/physical chemical analysis of glass dust, metal, wax, paint and paper (one paper on each).

Of the technical sub-specialisms, by far the largest number of papers in this area are dedicated to *ballistics*. Of these, 20 papers deal with different methods for determining firing range, making this a major thematic focus in this area. The second large group is made up of 11 publications on firearm identification from firearm trace evidence. There are just single publications on determining bullet trajectory in long-range shots and on evidence on the firing hand.

The objectives of forensic *writing examination* are to determine the authenticity or otherwise of a document and to identify the person responsible for a piece of handwriting or typescript. Nine papers are concerned with identifying an author based on individual handwriting features. Three papers look at criteria which enable handwriting and typescript to be falsified. One paper looks at typewriter identification and one at determining the age of a piece of handwriting or typescript. There is also a critical analysis which calls for proof of competence to be required before a court accepts the evidence of a writing expert (27/364).

The publications on *document examination* are concerned with the examination of paper scraps, betting slips and paintings.

One of the main areas of application of *dactyloscopy* is the comparative examination of fingerprints to identify individuals. Five papers describe the conditions required and evaluation criteria for fingerprint identification. There are 3 articles (10/372, 22/54, 27/323) on methods for visualising latent fingerprints. Two articles examine fingerprint identification in dead bodies in an advanced stage of decomposition (13/256, 29/426).

The field of *shape traces* deals with various impressions on a wide range of media. The publications in this field deal with tool, shoe and bite impressions and methods for preserving a trace.

In the field of forensic *photography*, it is essential that images are informative and authentic, as they constitute evidence admissible in court. The publications in this field therefore deal with photographic and technical requirements for producing usable photographs.

The sole work on *odorology* is a case report on the evidentiary value of olfactory traces of specific wood shavings (6/1).

The papers on *investigative techniques* deal with the application of UV light, spectrography and X-rays.

### Clinical legal medicine

There are two papers on *age estimation* in living subjects, one exploring diagnostic criteria in minors and one exploring criteria in adults. Leningrad author W. A. Nadeshdin produced a detailed discussion of features of the face and dentition useful for age estimation in adults (6/121). In a second work on age estimation in minors, he proposes growth, tooth eruption and signs of sexual maturity as a reliable combination of features for this purpose (8/273).

The papers analysed include just 2 early reports on *drug addiction*. The first, a case study (12/285) on multiple drug use with a preference for chloroform in a pharmacist, was published in 1928. The second report (35/17) from 1941 discusses the medical history of a patient who, following morphine addiction, had developed pethidine addiction.

A detailed discussion of the legal and social significance of *child abuse* (13/159) is the only publication on this subject.

The investigation of *self-inflicted injury* always aims to reveal the motivation. The 2 published cases are both cases of attempted insurance fraud, in one by severing the left thumb (23/352), in the other through many years of self-harm involving repeated injury to the left arm (38/244).

### Forensic psychiatry and psychology

The vast majority of publications on this topic are concerned with *psychopathology*. Of these, most are concerned with individual forensically significant psychopathological phenomena, discussed in 26 articles. There are 11 papers specifically on the forensic significance of psychiatric disorders. These include various psychoses, paralysis and dementia. Four case studies consider previous trauma in relation to the assessment of psychotic disorders. There are 3 papers on the principles and diagnostic value of graphology. There are a further 3 case reports on the psychopathology of prominent figures, including the sensational case of railroad killer Szilveszter Matuska (20/53). Of a paper by Roland Freisler, then Secretary of State in the Reich Ministry of Justice, entitled “The Psychology Underlying Polish Atrocities, Illustrated through the Development of the Polish National Character”, only the title was reproduced (34/7). The paper takes a social psychology approach, and the full text was published in a legal journal.

A themed group of 13 papers covers various subjects in the field of the *psychology of testimony*. Topics include the impairment of memory capacity, in particular through hypnosis and suggestion, recognising false testimony and assessing testimony given by children and adolescents.

The interdisciplinary field of *suicidology* is, in the selected context of forensic psychiatry and psychology, reduced to problems in this area. The publications classified as belonging to this field are therefore primarily concerned with motives and risk factors for suicide. There are 14 articles on these topics, which, in addition to the sequelae of diseases and head injuries, discuss individual constitution as a causal factor. Epidemiological surveys on suicide in Russia, in Finland and in Berlin are the subject of 3 papers. In a paper entitled, “Contribution on the Psychology of Suicide” (12/346), the phenomenon of imitation is discussed in the context of a popular suicide location.

### Sexual medicine

Of the many and varied references to human sex life subsumed into the category sexual medicine, one extensive group is the *paraphilias*—sexual perversions in the nomenclature of the time. Topics covered by the 23 publications in this area range from sexually motivated self-mutilation, to rare potentially criminal forms such as fetishism, to clearly criminal acts such as incest and paedophilia. There is also one article calling, as part of a pending reform of criminal law, for the abolition of Sect. 175 of the German Criminal Code (StGB), which at the time made consensual sexual acts between men a criminal offence. The first appeals for sterilisation for deviant sexual behaviour were published as early as the 1920s. The earliest work on “Castration and Sterilisation as a Means of Treatment” (3/162) from 1924 is concerned with applying these measures both in the event of a “mental disorder triggered by functions of the sexual organs” and for the elimination of “a criminal activity”. A later article (14/432) from 1930 rejects the idea of surgical castration of sex offenders, but advocates radiation treatment of recidivist sex offenders. These two publications are followed by 9 further papers, all relating to the “Law against Dangerous Habitual Criminals and on Measures of Reform and Prevention” (*Gesetz gegen gefährliche Gewohnheitsverbrecher und über Maßregeln der Sicherung und Besserung*) of November 24, 1933 and in particular to the newly introduced measure “castration of dangerous sex offenders” in Sect. 42a (5) of the German Criminal Code (StGB). The last of these papers, entitled “Observations on Castrated Sex Offenders” (33/248) concludes with the assertion that, “the success of castration has far exceeded expectations.” Two papers discuss the physical consequences of castration, and a further 2 papers fertility in the context of the Law for the Prevention of Offspring with Hereditary Diseases (*Gesetz zur Verhütung erbkranken Nachwuchses*).

There are 7 papers on *hymen examination*. These publications illustrate the extraordinary variability of the healthy hymen and to an even greater extent the pathologically altered hymen. Depending on shape and elasticity, where sexual violence is suspected, consideration must be given to the possibility of vaginal penetration without rupture of the hymen (14/265).

Fatal sexual activity without a sexual partner is the common element uniting the phenomenon of *autoerotic fatalities*. Classification of a death as an autoerotic fatality is solely dependent on the circumstances at the site of discovery and is independent of the cause of death. Of 6 cases reported, 3 were deaths by hanging, and of the remainder the cause of death was only able to be determined in one case.

Other sexual medicine topics are treated in 3 papers on male *sexual dysfunction* and 2 publications on *intersexuality*.

### Traffic medicine

Almost all publications in this field are concerned with the causes of and consequences of injuries incurred in *road traffic accidents*. These include 7 papers on the biomechanics of road traffic accidents. Injuries to the head and extremities are discussed with respect to their value for investigative purposes. Two papers discuss trace evidence after road traffic accidents and their use in accident reconstruction. A further 7 articles are concerned with the effects of alcohol on driving performance and the resulting accident risk. Two papers are concerned with other causes of accidents. Just one publication, titled accordingly, discusses the important question in *rail traffic accidents* of whether a victim was “Alive or Dead when Run over by a Train” (25/147).

### Social medicine

There are just 11 publications relating to social medicine. Seven articles are primarily concerned with *occupational problems* relating to diseases with social sequelae. There are also 3 articles concerned with public health and a positively classic social medicine paper entitled “The Effect of Social Situation on the Health Status of Welfare Recipients” (20/535).

### Criminology

Of the sub-specialisms historically forming part of the field of criminology, 19 publications on *criminal biology*, originally known as criminal anthropology, make it the best represented. Six papers in the then emerging research field of “blood groups and criminality” (9/426) form part of a series with 6 other papers on constitutional biology, most of which are concerned with identifying physical signs of a tendency to criminal activity. A prime example of forensic genealogical research is a case study entitled “A Criminal Family” (25/7). The increased focus on criminal biology, while pre-dating 1933, reaches its climax in the Nazi era. On this subject, Theodor Viernstein, head of the *Bayerische kriminalbiologische Sammelstelle* (Bavarian Biological Criminality Detention Centre) wrote, “The Law for the Prevention of Offspring with Hereditary Diseases of July 14, 1933 and the Law on Measures against Dangerous Habitual Criminals of November 23, 1933 are, for our question, two decisive landmarks on this road to the renewal of the living race of our people. What until the seizure of power remained, in the minds of scientists, psychiatrists, hereditary biologists and racial hygienists, a mere hope for the future, was given, at the very moment that the people faced their greatest hardship, practical form and legislative expression” (26/3).

There are 12 publications in the field of *criminal psychology*. In addition to a review on the concept and scope of the discipline from a 1940s perspective (36/119), there are 9 articles dealing with the psychology of specific offences, the most common of these being arson, to which 4 articles are dedicated. Finally, there are 2 papers discussing crimes committed under hypnosis.

*Criminal phenomenology* encompasses publications on the phenomenology of various groups of offences. The only group on which multiple papers are published is youth criminality. There are 5 papers on this subject, in one of which the supposedly modern term “hooliganism” is used in a wide-ranging sense (12/361). Heroin misuse, already widespread in the mid-1920s, is used to illustrate the consequences of drug addiction (8/81). Other topics include homicide, sexual offences and arson. The 3 papers in the field of *victimology* look at the physical and psychological sequelae experienced by female minors who have been the victim of a sexual offence. There are 2 publications on *crime prevention*, both discussing the basic idea that social welfare is an important component of crime prevention. In the *criminal aetiology* field, there is one article on the effects of war on criminality (1/697) and another on “Reading as a Stimulus to the Commission of Crime” (13/209).

Of 20 publications on *prisons and imprisonment*, 8 concern self-harming by prisoners. The incidents described range from inducing symptoms of illness and swallowing foreign objects, to significant self-mutilation. A number of papers are also dedicated to the topic of illness and its treatment in prisoners. The 6 articles on this issue are primarily concerned with physical illness. Four papers tackle the subject of the role of medical personnel in prisons. Two further articles discuss the particular problems faced by adolescents in prison (4/121) and support for prison leavers in Germany (11/423).

## Discussion

The period under review commences in 1922, shortly after the establishment of the Weimar Republic, and ends in the spring of 1944, in the final phase of the Second World War. The Nazi seizure of power in early 1933 represents a profound political and historical rupture. For legal medicine, a discipline with close links to the state, this social upheaval inevitably had an impact on issues and outlooks within the discipline. A comparison of the 19 volumes published from 1922 to 1932 with the 19 volumes published from 1933 to 1944 shows that, in addition to the expected effects on legislation and jurisprudence, there was also a transformation in the sociological and even in some cases medical topics within the discipline. Particularly striking is the link between legal medicine and the Nazi creed of “*Erb- und Rassenpflege*” (“genetic and racial hygiene”).

The start of the period under review saw legal medicine as a discipline attains a long-awaited goal. Following repeated calls from prominent experts in the field (2/75, 19/264), in 1924 the discipline was finally included in the state medical examination, having been a compulsory subject for students of medicine since 1901 [11]. On the question of how teaching should be organised, there was little agreement across German universities. With the exception of the core field of anatomy, teaching had developed in two quite different directions. One emphasised the psychological and psychiatric side of the discipline, an approach promoted by, for example, Willy Vorkastner in Greifswald and Heinrich Többen in Münster. The other favoured a scientific-criminalistic approach, promoted by, for example, Richard Kockel in Leipzig and Theodor Lochte in Göttingen. Under the influence of Nazi higher education policy, the integration of criminalistic content determined the profile of the discipline. The visible expression of this development was the introduction in 1940 of a standard designation, “Institute of Legal Medicine and Criminalistics” [12], across the Reich, and the increasing amount of work that was not strictly medico-legal in nature.

Articles in the *Deutsche Zeitschrift* discuss a number of pieces of legislation which emphatically highlight the consequences of Nazi ideology for the judiciary. While the vast majority of laws were rendered legally void by Control Council Law No. 1—Repealing of Nazi Laws of September 20, 1945, the provisions introduced in 1934 by the Law against Dangerous Habitual Criminals and on Measures of Reform and Prevention—with the exception of the provision on castration in Sect. 42a of the old version of the German Criminal Code—remained largely unchanged. The provisions on preventive detention were incorporated into the Criminal Code in 1953 (BGBl. I p. 735) and tightened in 1969 (BGBl. I p. 645). As a result, the Law on Habitual Criminals introduced a dual system of sanctions into German criminal law which still exists today.

At the 11^th^ congress of the scientific association held in Erlangen in 1921, the main topic was the introduction of coroners autopsies. Three speeches were dedicated to the reasons why it was necessary to have this option available (1/1, 1/9, 1/12). This unanimous appeal by all three speakers was further reinforced by the speech which followed them, entitled “Experiences with Coroners Autopsies in Hamburg” (1/17). Despite the endeavours of the scientific association, no uniform legislation on this was forthcoming, with the result that in 1936 calls for the introduction of coroners autopsies were again the topic of the opening speech (28/1) at the 25^th^ congress in Dresden. The text drafted by speaker Hermann Merkel entitled, “Reich Legislation to Introduce Coroners Autopsies” was unanimously adopted by congress participants. Despite this, corresponding legislation was still not on the statute books by the end of the war. It was not until 1949 that legislation was passed in East Germany to enable coroners autopsies to be performed in cases where the cause of death was unclear. Forty years of positive experiences [13] did not, however, lead to the adoption of a uniform legal framework for coroners autopsies in the Federal Republic of Germany.

Post-mortem rectal temperature measurement has proven to be highly valuable for estimating time of death. The first studies on the rate of cooling of dead bodies were published in 1937. Successive rectal temperature measurement produced very regular exponential decay curves. The temperature initially drops only modestly, followed by a period in which it drops more quickly, followed by a further period of slower decline (28/172, 29/158). Though first described many years ago, this sigmoidal cooling curve still forms the basis of standard methods of temperature-based time of death estimation even today [14].

Through a case report on the examination of an exhumed skull (8/430), Kurt Walcher drew expert attention to the rule on the sequence of skull fractures first published by Georg Puppe a few years earlier. Evaluation of fracture lines showed that the victim had suffered at least one hard blow before being killed by a gunshot to the head. As in the above case, it has subsequently proved possible to use this principle to determine the sequence of multiple skull fractures, including fractures arising through different means. Puppe’s rule is now regarded as essentially proven [15].

A wide variety of views have evolved on the mechanism of coup-contrecoup brain injuries. Lenggenhager [16] theorised that blunt force at the point of impact where the brain flattens against the skull causes blood and cerebrospinal fluid to be squeezed out of the inside of the skull. At the point opposite the impact, a severe reduction in pressure—in severe cases even a vacuum between the cranium and the brain—can occur. This causes the characteristic pressure differential or suction haemorrhage in the contrecoup area. Shortly after publication Carl Franz entered into a debate with its originator in the pages of the *Deutsche Zeitschrift*. Franz was of the opinion that the theory must be wrong, as the speed of the process meant that it was not possible for the posited outflow of blood and cerebrospinal fluid to occur (31/61). In his reply (31/278), Lenggenhager explained his theory using his original publication. Though in his concluding remarks (31/280) Franz maintained his objection, the suction theory is today considered the most scientifically reasonable explanation for contrecoup injuries [17, 18].

By the early 1920s, there had already been a number of works on the mechanism of death by hanging. Nonetheless, it remained unclear “to what moment—whether it be the closure of the airways, the obstruction of the cervical vasculature or the effect on the vagus nerve—is to be attributed the main role in death by hanging” (1/686). Georg Strassmann studied the effect of strangulation on the airways and found that, for normal hangings, it was impossible to overcome the obstruction of the airways by even “the most strenuous inhalation or exhalation”. Where the noose was applied in an atypical position, it appeared that there was at the least “greater difficulty in obtaining air” (4/165). Walther Schwarzacher performed experiments to determine the tensile force required to obstruct the cervical vasculature (11/145). With a standard symmetrical noose position and a constant internal vascular pressure of 170 mmHg, a force of 3.5 kg is sufficient to obstruct the common carotid arteries, while a compression force of 16.6 kg is required to obstruct the vertebral arteries. The results of these two series of experiments remain part of the corpus of knowledge on the mechanism of death by hanging even today.

Techniques for diagnosing death by drowning were enriched by the repeated observation by Erich Fritz of a specific morphological finding (18/285). He identified tears in the mucous membrane of the stomach in several victims of drowning in which there was no evidence of any violence to the body prior to drowning. With respect to the origin of this indication of drowning, after some initial uncertainty the opinion prevailed that the tears are caused by overstretching of the stomach primarily through swallowing the drowning medium [19]. Gyula Balázs presented a collection of case studies on “Unusual Skin Findings Following a Fall into Water” (21/515). This paper contains the first description of “ischaemic impact patterns”, also of diagnostic value in the form of a double stripe on skin beaten with a stick.

As with the numerous forensic science publications on ballistics, forensic traumatology papers have similarly enriched our understanding of the effects of gunshots. This is particularly true for the first paper by Anton Werkgartner in the *Deutsche Zeitschrift* on muzzle imprint in contact wounds (11/154). A muzzle imprint proved to be a reliable sign of a contact wound, and its characteristic shape can also provide information on the type of weapon and the position of the weapon on firing [20].

In the forensic obstetrics field, most publications are concerned with questions arising in the context of Sect. 90 of the German Code of Criminal Procedure (StPO). The main focus here is on determining whether an infant was alive at birth. In addition to the lung float test, for the abolition of which Albin Haberda appeals in a piece unequivocally entitled, “Out with the Lung Float Test!” (14/7), János Prievara presented the cord vessel test (36/21) after Jankovich [21] as an additional method of determination. The test is based on the principle that the umbilical arteries in stillborn babies remain open and are therefore permeable to water, whereas the umbilical arteries in babies which are alive at birth do not. In the author’s practical experience, a positive result from the umbilical vessel test is a very reliable supplement to standard tests for live birth.

There are various, generally non-specific signs on a body that may lead to a suspicion of poisoning. In some cases, specific pathological anatomical changes may act as an initial indication of potential poisoning. In a paper entitled “Post-mortem Findings Following Sedative Poisoning” (34/307), Franz Josef Holzer reported his systematic observations on the topic. He identified characteristic violet patches, often symmetrical, especially in particular areas on the legs. These lesions, today known as coma blisters, are observed particularly in barbiturate poisoning.

In 1932, almost contemporaneously with the German edition of Erik M. P. Widmark’s ground-breaking monograph [22], the *Deutsche Zeitschrift* carried a paper entitled “On a Technique for Quantitative Determination of Blood Alcohol Levels after Widmark’s Method” (19/513). Validated by numerous studies, Widmark’s micromethod established itself as the standard technique for determining blood alcohol.

Over the period under review, advances in methodologies for the identification of unknown bodies occurred primarily in the fields of anatomy, anthropology and osteology. In addition to publishing an article on plastic reconstruction of the physiognomy (8/365), anatomist Friedrich Stadtmüller also subsequently developed a graphical method for verifying skull identification (20/33, 27/335) based on Welcker’s method [23]. Dionys Schranz’s extensive study on identifying features of the humerus (22/332) contains ground-breaking data. His results on age estimation in particular are still standard criteria today [24].

Following the discovery of the main blood groups in 1901, the discipline of forensic serology developed into a more and more significant field [25]. In the period under review, scientific interest was focused, in addition to AB0 blood groups, on A-subgroups and the MN system. This enabled the gradual resolution of key problems relating to the use of blood groups in trace evidence analysis and in family relationship evaluation. Lattes’ coverslip method (9/402) and Schiff and Holzer’s agglutinin binding test (16/445) became established methods for the individuation of bloodstains in the 1920s. Sufficiently reliable methods were also available at the time for other questions arising during the examination of bloodstains. Peroxidase and crystal tests were available for detecting blood, and the now well-established Uhlenhuth test was available for determining species. For family relationship evaluation, genetic and serological test methods would continue to coexist for some time. Although a 1926 review by Fritz Schiff stressed the significant advantages of blood groups over morphological characteristics (7/360), it was not until serological testing was able to rule out the paternity of a significantly higher proportion of non-fathers that it became the dominant method.

The scientific disciplines of forensic technology have the greatest overlap with legal medicine, both in theory and in practice. Consequently, legal medicine specialists have published numerous scientific articles and case reports on forensic biology and forensic chemistry. The publications evaluated here include significant excursions into the quite separate field of criminalistics, for example papers on traces of blood in snow (31/213) and the analysis of incendiary agents (36/245). Of the scientific sub-disciplines, the most prominent is ballistics, on which there are many papers which belong thematically to the field of criminalistics. This is particularly clearly illustrated by publications on firearm identification from trace evidence on munitions (18/350) and on cartridge cases (21/190). Similarly, numerous publications on writing examination can without exception be filed under forensic technology. Martin Nippe’s call for legal medicine institutions to take over criminalistic tasks (14/411) reflected a contemporary imperative aimed at providing greater legal certainty, as there were simply not enough experts in forensic technology. In the long term, however, legal medicine needed to be liberated from such a non-medico-legal role, and this occurred only gradually in the post-war period.

Clinical legal medicine is significantly underrepresented compared to more recent times. There are only a handful of publications on the subject, and there are no papers on a number of significant health-related issues. Particularly notable by their absence are articles on the physical examination of victims and perpetrators of violent and sexual offences. Child abuse is another area which is notably underrepresented, with insufficient consideration of the complex fabric of causation and the many different patterns of harm. In an extensive analysis (13/159), Ernst Ziemke laments the fact that “reports from experienced legal medicine specialists and my own experience suggest that it is relatively rare for cases of child abuse to be subject to a medico-legal assessment. Where they are, this is almost always cases in which the outcome of the abuse has been the death of the child.”

The publications analysed here deal with a wide range of forensic psychiatry and psychology topics. Legal reforms and the effects of changes to our understanding of medical psychology mean that the technical content has changed repeatedly since. Terms such as neurosis (1/325) and hysteria (2/523) are now considered outdated, and significant progress has been made in areas such as intelligence testing (8/580). The psychology of testimony, one of the cornerstones of interrogation theory, has evolved into an extensive sub-specialism within the forensic psychology field.

In sexual medicine, there has been a quite contradictory development. While the sexology side was working towards liberalisation, controversial legislation on sexual offences remained in place. The quite reasonable call for the repeal of Sect. 175 of the German Criminal Code (StGB) (13/59) was not heeded until the end of the war. Even more draconian were the newly introduced “Measures of Reform and Prevention”. Under the 1933 Law on Habitual Criminals, a court could order, under Sect. 42a (5) of the Criminal Code, the “castration of dangerous sex offenders”. As long as certain statutory conditions were met, Sect. 42 k (1) of the Criminal Code extended the groups covered by this provision to include sex offenders with repeated convictions for exhibitionism. While there were many publications on the draconian measure of castration and its consequences, there is by contrast a notable absence of publications on sexual therapy for the afflicted.

Increasing motorisation of road traffic saw the emergence of traffic medicine as a new sub-specialism within the legal medicine field. Publications on road traffic accidents are concerned with either the causes of accidents or the consequences of injuries. With the advent of motorised road traffic, it was not long before alcohol consumption by drivers (29/11) and pedestrians (32/312) was identified as a notable cause of accidents. There followed experimental studies aimed at obtaining concrete data on how alcohol affects driving performance, for example the effects of alcohol on eye and ear function (32/301). Results from these studies also represented an academic foundation on which to base road traffic legislation. With respect to the consequences of injuries, there was a need to clarify common patterns of injury and their origin (24/379, 33/124). Insights gained into mechanisms of trauma offered an opportunity to use medico-legal findings to contribute to accident reconstruction.

Given that for many years the name of the German scientific association included the words “social medicine”, the paucity of publications in this field is particularly surprising. The small number of works present are not even vaguely representative of the interdisciplinary field of social medicine.

In keeping with the concerns of the period, most of the papers in the field of criminology are related to criminal biology. Studies of offenders inspired by constitutional biological investigations claimed to find features characteristic of born criminals and thereby to justify a robust approach to the fight against crime. In addition to studies on morphological features, efforts were also made to identify serological markers. A significant field of research at the time was the relationship between blood groups and crime. Results from such studies were, however, somewhat contradictory. In a study on more than 1000 convicts, Kurt Böhmer found “a general increase in blood group B. This increased further in recidivists and the irreformable” (9/426). Augustin Foerster’s accurate conclusion unambiguously disagreed. He was unable to confirm “that blood group B is more common in the prison inmates I examined. It was not possible to demonstrate any link between blood group and criminality” (11/487). A key area in criminal psychology is arson, the explanation for which in one case shifted from the classic pyromania to a “simmering subconscious sexuality” (33/52). To obtain usable insights into significant phenomenologies of criminality, criminal phenomenology requires up-to-date studies. Only if this condition is met can results from studies be used to formulate policy on crime or be applied to forensic science investigations. For this reason, publications on a range of phenomenological criminology topics are of historical interest only. Articles on female minors who have been the victim of a sexual offence represent the beginnings of victimology, which evolved into an academic discipline only after the Second World War.

In conclusion, it is worth noting that, despite the many papers from a variety of fields closely allied with but distinct from legal medicine, most of the published papers are concerned with the traditional key fields of traumatology and toxicology. There are also large numbers of articles on various topics in the thanatology and forensic obstetrics fields. An emerging sub-specialism is the study of blood groups, which found increasing use in trace evidence analysis and in family relationship evaluation.

## Footnotes

1 The first number refers to the volume, the second to the start page.
